# Effects of Modified Ramadan Fasting on Mental Well-Being and Biomarkers in Healthy Adult Muslims — A Randomised Controlled Trial

**DOI:** 10.1007/s12529-024-10296-0

**Published:** 2024-05-22

**Authors:** Romy Lauche, Iman Fathi, Chalil Saddat, Petra Klose, Jallal Al-Abtah, Arndt Büssing, Thomas Rampp, Gustav Dobos, Holger Cramer

**Affiliations:** 1 National Centre for Naturopathic Medicine, Southern Cross University, Military Rd, Lismore, NSW 2480 Australia; 2 Department of Internal and Integrative Medicine, Faculty of Medicine, Evang. Kliniken Essen-Mitte, University of Duisburg-Essen, Essen, Germany; 3 Institute of Integrative Medicine, Faculty of Medicine, University of Witten/Herdecke, Herdecke, Germany; 4 Centre for Complementary and Integrative Medicine, University Hospital Essen, Essen, Germany; 5 Institute of General Practice and Interprofessional Care, University Hospital Tübingen, Tübingen, Germany; 6 Bosch Health Campus, Stuttgart, Germany

**Keywords:** Ramadan, Religion, Fasting, Nutrition

## Abstract

**Background:**

Ramadan fasting has seen increased attention in research, often with inconsistent findings. This study aims to investigate whether dietary and lifestyle modifications during Ramadan can improve well-being and health in healthy adult Muslims.

**Method:**

A randomised controlled trial with two parallel groups was conducted in an outpatient clinic of a university hospital in Essen, Germany, in 2016. Healthy adult Muslims (*n* = 114) aged 18–60 years were randomised to a modified fasting group; i.e., they received educational material prompting dietary and lifestyle modifications pre-Ramadan, and a control group who undertook Ramadan fasting as usual. Primary outcome was quality of life (*WHO-5 Well-Being Index*). Secondary outcomes included sleep quality, spirituality, and mindfulness (all self-report), body weight, body mass index, body fat, waist circumference, hip circumference, blood pressure, and heart rate, as well as blood serum biomarkers. Safety was examined via adverse events.

**Results:**

The modified fasting group reported significantly higher quality of life (WHO-5) compared to the control after Ramadan (MD 5.9; 95% CI, 0.02–11.8; *p* < 0.05). Group differences in favour of the modified fasting were also found for satisfaction with health (MD 5.9, 95% CI 0.19–11.67), ease of life (MD 4.1, 95% CI 0.38–7.80) and mindfulness (MD 7.6, 95% CI 2.68–12.52), reductions in weight (MD, − 0.9 kg; 95% CI − 1.39 to − 0.42), BMI (MD − 0.3 kg/m^2^, 95% CI − 0.50 to − 0.15), hip circumference (MD − 0.3 kg/m^2^, 95% CI − 0.50 to − 0.15), and diastolic blood pressure (MD − 2.8 mmHg, 95% CI − 5.15 to − 0.43). About 60% of participants reported adverse events, mostly headaches/migraines, dizziness/fatigue, common cold, and gastrointestinal symptoms, with no group differences. One serious non-related adverse event each occurred in both groups.

**Conclusion:**

Pre-Ramadan dietary and lifestyle advice can lead to short-term improvements in mental and physical well-being of adult Muslims observing Ramadan. As such, this study demonstrates the potential benefits of culturally appropriate health interventions in a religious context.

**Trial Registration:**

ClinicalTrials.gov (Identifier NCT02775175).

## Introduction

Fasting during the month of Ramadan is one of the five pillars of the Islamic religion and a religious requirement for all healthy Muslim adolescents and adults [1]. Ramadan fasting dictates self-restraint from food and drink, as well as from medications, smoking, and sex, between dusk and dawn encompassing a period of 10–21 h/day for 29–30 consecutive days, depending on the alignment of the Islamic lunar calendar with the climate and season at a given geographical location [2]. Ramadan is an opportunity for contemplation, prayer, and other spiritual activities [1, 3]; the practice of Ramadan however differs greatly around the world by virtue of the surrounding culture, socioeconomics, diet patterns, and spiritual approaches among different populations [3, 4]. Studies on the effects of Ramadan fasting produce conflicting and inconclusive results for body composition, biochemical markers, pregnancy, and disease-related outcomes [5–13]. Potential explanations for this include variations in local climates and seasons (influencing fasting hours and activities), study design, baseline health of participants, and sample collection [3]. More importantly, it can be assumed that dietary factors play an important role, with some studies observing increased sugar and carbohydrate consumption, higher overall caloric intake, overeating at night [14–20], and lower water/fluid intake [21, 22], in conjunction with reduced physical activity [23, 24] and reduced duration and quality of sleep [11, 25–27] during Ramadan.

While Ramadan fasting has seen increased attention in research [28–31], most research on the potentially detrimental effects of Ramadan fasting has been conducted with at-risk groups, such as pregnant women, elite athletes, and those with chronic disease (such as diabetes and kidney disease) [5, 29, 32–46]. In addition, previous Ramadan research in healthy adults has primarily focused on physical and biochemical health indicators (e.g. body mass [7, 12, 30, 47, 48], blood pressure [5, 32], and blood lipid parameters [49, 50]; glycaemic control [2, 51]; endocrine [28, 52], liver [53], and kidney function [54]; sports performance [44, 55, 56]; and birth metrics [41, 57]). While there is growing evidence on the influence of diet and nutrition on mental health and well-being [58–60], well-being parameters have rarely been incorporated into Ramadan research, specifically in relation to dietary patterns. As such, there is a clear need to understand whether dietary changes during Ramadan affect other dimensions of health (mental health, spirituality, quality of life, and overall well-being).

The current trial aims to close this gap and investigate the effects of an intervention targeting dietary and lifestyle factors on quality of life and well-being during Ramadan for the first time. Due to the mandatory nature of Ramadan fasting, observers cannot be randomised into participating/non-participating; therefore, previous studies typically lacked a true control group, and comparisons were typically made between pre-, during-, and post-Ramadan sampling points [7, 12, 44, 61–63]. To overcome this, a pre-Ramadan dietary and lifestyle intervention was developed for implementation in one group to enable a comparison to a control group, which conducted Ramadan fasting as usual. Such study might provide valuable insights into the benefits of an add-on intervention to a religious practice, specifically for observers who feast on high cholesterol, high sugar, and processed foods [19] and adopt other unhealthy nocturnal behaviours during Ramadan causing potentially detrimental effects [3, 22].

Therefore, this study aims to determine whether a modified healthy fasting regimen may be beneficial for quality of life, and well-being of adult Muslims undergoing the 2016 Ramadan in Germany.

## Methods

### Study Design

This study used a RCT design with parallel groups and a 1:1 allocation ratio. The trial site was the outpatient clinic of the Department of Internal and Integrative Medicine, Evang. Kliniken Essen-Mitte, a teaching hospital of the Medical Faculty, University of Duisburg-Essen, in Essen, Germany. The trial was conducted between May and December 2016, with recruitment commencing between May and June 2016. The Ramadan period began on 6 June 2016 and ended on the evening of 5 July 2016. The trial protocol was reviewed and approved by the Ethics Committee of the Medical Faculty at the University of Duisburg-Essen (approval number 15-6336-BO). The trial was registered with ClinicalTrials.gov (Identifier NCT02775175).

### Participants

Participants were recruited via advertisement in Muslim community centres in and around Essen. After preliminary telephone screening, eligible participants were invited for further assessment by the study physician. The physician provided detailed study information and, after obtaining written informed consent, checked the participants’ medical history, and determined their overall health status and eligibility for participating in the trial.

Participants were deemed eligible for inclusion if they were aged 18 to 60 years, were physically and mentally capable of fasting, had already committed to observing the 2016 Ramadan, and had observed Ramadan fasting in the past. Exclusion criteria included untreated or malignant hypertension, diagnosed psychological conditions (depression, schizophrenia, addiction, eating disorders), severe disorders such as diabetes mellitus, cancer without remission, and rheumatic diseases. Further exclusion included women during pregnancy or breast feeding, people currently dieting, or those with a body mass index (BMI) < 20 or > 40.

### Sample Size Calculation

Using G*Power Software [64], we based our sample size on the following deliberation: in order to detect a moderate group difference on the primary outcome (effect size Cohen’s *d* = 0.5), 51 participants per group would be sufficient using a one-sided *t*-test with a significance level of *α* = 0.05 and a power of 1-*β* = 0.80. Accounting for a loss of power due to a maximum of 10% dropouts, we planned to include 57 participants per group; i.e., a total sample size of 114 was aimed for.

### Randomisation and Masking

Participants were allocated to one of two groups in order of appearance adopting a computer-generated (Random Allocation Software, version 1.0.0) [65] block randomisation with randomly varying block sizes. A stratum was applied for the participants’ sex. The trial coordinator, who was not involved in participant recruitment or outcome assessments, generated the list, and prepared sealed opaque envelopes with randomisation assignments; envelopes were labelled according to the study participant’s sex and ID number to be opened in ascending order by the study physician after baseline measurement. The study physician was not involved in participant outcome assessments.

### Masking

Neither participants nor the study personnel were blinded to the intervention. However, the outcome assessors were blinded to the group allocation at 4 weeks and 6 months when handing out/collecting the questionnaires and taking physical measurements. The laboratories employed to analyse blood samples also did not receive any information on the trial assignment of participants.

### Procedures

#### Intervention Group (Modified Fasting)

The intervention group received an educational booklet specific to Ramadan fasting. The booklet contained information about the history and background of Ramadan, the general effects of fasting on physical and mental health, and diet and lifestyle modifications that might be beneficial for overall health and well-being during Ramadan. It also contained recipes for healthy wholefood meals. This advice was partially based on a brochure entitled *Ramadan Health Guide - a guide to healthy fasting* published by Communities in Action with support of the National Health Services (NHS) in the UK [66].

More specifically, the booklet advised participants to consider and reflect on the quantity of food consumed, and to avoid overeating at night. It also provided guidance on and recipes replacing unhealthy foods with healthier alternatives (e.g. to enjoy light meals such as salads and soups over high-caloric meals such as meat and pasta, and to choose wholefood, home-cooked meals over processed, take-away food). It was suggested that they reduce intake of sweet, fatty, and salty/spicy foods, and prioritize a balanced diet with plenty of fruit and vegetables. The booklet highlighted the importance of keeping well-hydrated with water and tea during the permitted time frame and suggested to avoid sugary or sweetened drinks. Participants were further encouraged to stay physically active, however, to adapt their exercise load to their individual fasting regimen and physical health. Finally, the booklet contained a question-and-answer section to discuss, and provide solutions for, common health problems during fasting. The intervention consisted of educational advice only; there were no compulsory components.

#### Control Group (Fasting as Usual)

The control group participants were asked to continue with their usual Ramadan diet and activities without any modifications. In fairness, they were provided with the same material as the modified fasting group upon completion of the study.

### Outcomes

Sociodemographic characteristics such as age, sex, marital status, educational level, and employment were collected at baseline, as was information on lifestyle factors (alcohol consumption, smoking, and physical activity). Participants were also asked about their motivation for fasting (religious, spiritual, health, cleansing etc.). Outcomes were measured before Ramadan (baseline), after Ramadan (week 4), and at 6 months (follow-up), except for biomarkers which were not assessed at follow-up.

#### Well-Being Questionnaires

Spiritual and mental well-being was assessed using the following questionnaires:

- The World Health Organization *WHO-5 Well-Being Index* was used to assess subjective quality of life [67]; it includes five items related to positive mood, vitality, and general interests in life [68].
- The 10-item *BMLSS-10* (*Brief Multidimensional Life Satisfaction Scale*) was used to measure satisfaction with life on five domains: intrinsic, social, external, perspective, and health [69].
- The *PROMIS*™ (*Patient-Reported Outcomes Measurement Information System*) *Sleep Disturbance* and the *Sleep Related Impairment* Short Forms were used to assess sleep quality and sleep disorders [70].
- The *SpREUK-P SF17* (*Spiritual and Religious Attitudes in Dealing with Illness – Frequency of Engagement in Spiritual Practices*) questionnaire [71] examines frequency of engagement in organized and private religious, spiritual, existential, and pro-social-humanistic practices.
- The 10-item *CPSC* questionnaire (*Conscious Presence and Self Control*) [72] was used to determine the level of ‘daily life mindfulness’ or situational awareness.
- Meaning, peace, and dimensions of a person’s spirituality were assessed using *the FACIT-Sp* (*Functional Assessment of Chronic Illness Therapy - Spiritual Well-Being Scale*) [73].
- Easiness of life was addressed with the Light Heartedness/Easiness and Social Interest/Contacts (LHE/SIC) Scale [74].
- The *DSES* (*Daily Spiritual Experience Scale*) was used to determine a person’s perception of the transcendence in daily life, which may reflect their general level of an experiential aspect of spirituality [75, 76].
- The *DUREL* (*Duke University Religion Index*) was used to examine organized and non-organized religious activities and intrinsic religiosity, which is used as a measure of religiosity [77]. Both the DSES and DUREL were only used at baseline and included in the analyses as potential confounders.

#### Anthropometric Measures

Anthropometric measures included height, weight, BMI, body fat percentage, waist circumference, hip circumference, blood pressure, and heart rate. Weight and body composition (body fat percentage) were determined using a standard bioelectrical impedance device (BF 511, OMRON Healthcare, Mannheim, Germany). Waist circumference was measured with a measuring tape positioned in the horizontal plane exactly midway between the iliac crest and the costal arch, using the mean score of two consecutive measurements [78]. Hip circumference was measured in the horizontal plain at the maximal circumference of the hips or buttock region above the gluteal fold, and two consecutive measurements were averaged. Blood pressure and heart rate were examined using a standard automated digital blood pressure monitor on the side with the higher blood pressure values, using the arithmetic mean of two consecutive measurements for analyses.

#### Blood Serum Biomarkers

Blood serum markers included triglycerides, cholesterol, HDL and LDL cholesterol, fasting glucose, AST (aspartate aminotransferase), ALT (alanine aminotransferase) and GGT (gamma-glutamyltransferase), uric acid, and creatinine. All participants were asked to present fasted for the blood drawing; i.e., the appointments were scheduled in the early mornings (before Ramadan), and in the evenings (within the last 2 days of Ramadan, before fast breaking and festivities). All haemal markers were determined by a certified and DIN EN ISO 15189:2014 accredited hospital laboratory in Essen, Germany.

#### Adherence and Experiences

Participants in the modified fasting group were asked to indicate to what degree they adhered to the advice given (in percent) after Ramadan. They were further asked which of the advice they felt was most suitable, and whether they followed it. An open-ended question also asked about their experiences with the dietary and lifestyle modifications compared to the last time they fasted.

#### Safety

Participants were asked to report any adverse events during the study period, even if perceived as insignificant (e.g. a common cold). Adverse events were defined in accordance with the ICH GCP guidelines as any untoward medical occurrence, i.e. any abnormal laboratory finding, symptom, or disease temporally associated with study intervention, whether or not caused by the intervention. All adverse events were recorded by the study coordinator and participants experiencing such events were asked to consult with the study physician to assess the severity and initiate any necessary response.

#### Primary Outcome

Quality of life according to the *WHO-5 Well-Being Index *after Ramadan (week 4) was defined as the primary outcome measure. All other outcomes were thus secondary.

### Statistical Analysis

Analyses were performed using the Statistical Package for the Social Sciences software (IBM SPSS Statistics for Windows, release 24.0. Armonk, NY: IBM Corp.) and were based on the intention-to-treat (ITT) population; i.e., every subject providing baseline data was included in the analyses. Missing values were substituted using the SPSS Markov chain Monte Carlo multiple imputation technique, where 50 new datasets were generated for each missing value and the average scores of those were used to replace missing values.

We used analysis of covariance (ANCOVA) to model the given post-treatment outcome as a function of the treatment group (classified variable), the respective baseline value (linear covariate), and confounding variables for which a significant group difference was found at baseline (covariates, e.g. age, gender, weight, spirituality, reasons for fasting, lifestyle factors such as alcohol consumption, smoking, diet). The *p*-value was set as 0.05 for the primary outcome. Secondary outcomes were reported without *p*-values. Categorical data were analysed using chi-square tests. Correlational analyses were also conducted to determine associations between outcomes.

## Results

### Participants

Of the 206 individuals initially screened by telephone, 122 were examined by the study physician and 114 were enrolled. Common reasons for exclusion were failing to meet the health criteria or discontinued interest in the study. Two of the 114 enrolled participants did not present for baseline and therefore were excluded; replacement was not viable due to the restricted baseline collection window before Ramadan commencement.

**Fig. 1:**
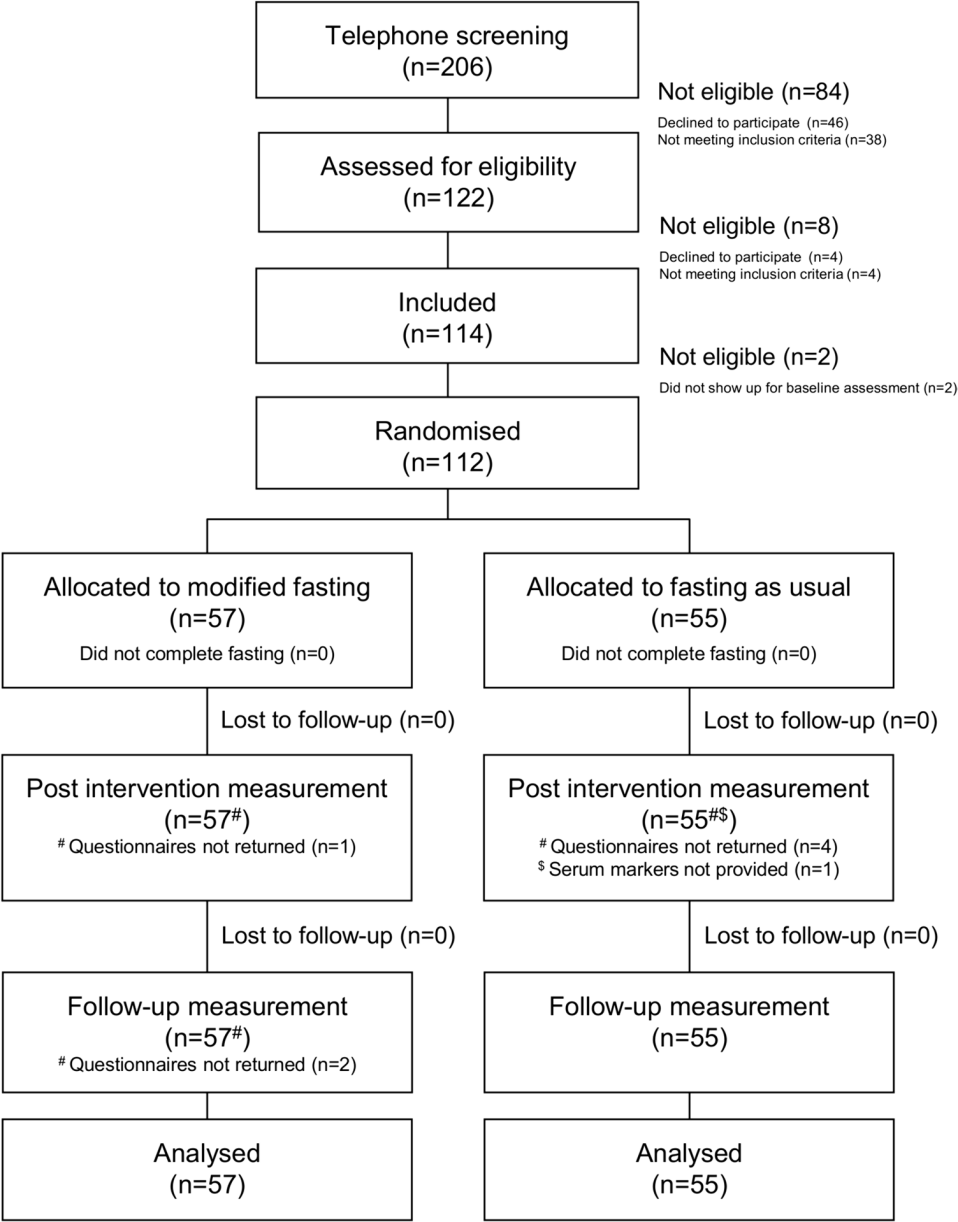
Consort flowchart of participant recruitment and study flow

No participants were lost throughout the study; however, some did not return their questionnaire (*n* = 5) or did not have their blood sample taken (*n* = 1) post-Ramadan; i.e., they provided partial data. At follow-up, two participants did not return their questionnaires, but anthropometric measures were taken. Data from 112 participants was analysed using intention-to-treat (Fig. 1).

### Baseline Characteristics

Participants were 27.8 ± 9.4 years of age on average (mean ± standard deviation); 63 were women and 49 were men (Table 1). Nearly two-thirds of participants were not in a relationship, while one-third were in a relationship or married. Most participants had a high school degree, and a substantial proportion were attending or had graduated from university.

**Table 1:** Baseline characteristics of trial participants according to treatment group

**Item**	**Modified fasting** ***n* = 57**	**Fasting as usual** ***n* = 55**
Age in years	28.4 ± 9.8	27.1 ± 9.0
BMI in kg/m^2^ (mean ± SD)	26.2 ± 4.3	25.6 ± 4.3
Sex female *N* (%)/male, *N* (%)		
Female	32 (56.1%)	31 (56.4%)
Male	25 (43.9%)	24 (43.6%)
Marital status, *N* (%)		
Single	35 (61.4%)	38 (69.1%)
In a relationship or married	19 (33.3%)	17 (30.9%)
Separated, divorced, or widowed	2 (3.5%)	0 (0,0%)
Education, *N* (%)		
< High school degree	10 (17.5%)	10 (18.2%)
High school degree (including those in higher education)	31 (54.4%)	34 (61.8%)
University degree	16 (28.1%)	11 (20.0%)
Employment, *N* (%)		
Unemployed (incl. home keepers)	18 (31.6%)	22 (40.0%)
Employed (part-time and full-time)	29 (50.9%)	26 (47.3%)
Retired	1 (1.8%)	0 (0.0%)
In training (university)	7 (12.3%)	5 (9.1%)
Alcohol, *N* (%)		
Abstainer	56 (98.2%)	54 (98.2%)
Low-risk drinking	1 (1.8%)	1 (1.8%)
Smoking, *N* (%)		
Not smoking	53 (93.0%)	48 (87.3%)
Smoking	4 (7.0%)	7 (12.7%)
Physical activity, *N* (%)		
No exercise	6 (10.5%)	7 (12.7%)
Low intensity	7 (12.3%)	6 (10.9%)
Moderate intensity	9 (15.8%)	6 (10.9%)
High intensity	35 (61.4%)	36 (65.5%)
Reasons for fasting, *N* (%) Multiple responses possible		
Religious obligation	55 (96.5%)	54 (98.2%)
Spiritual reasons	43 (75.4%)	42 (76.4%)
Health reasons	19 (33.3%)	14 (25.5%)
Physical and mental cleansing	43 (75.4%)	40 (72.7%)

The ethnic origins of participants were diverse, with participants mainly from Turkey (*n* = 34), Morocco (*n* = 32), and Afghanistan (*n* = 15), but also Palestine, Lebanon, Germany, Iran, Iraq, Syria, Bosnia, Greece, Italy, India, Pakistan, Sri Lanka, and Chechnya. All but one participant chose to abstain from alcohol, and less than 10% were smoking. Over half of participants reported a high level of baseline physical activity. Regarding motivations for fasting, almost all participants fasted due to religious obligation; however, many also indicated that they were motivated by spiritual reasons and physical/mental cleansing.

### Well-Being Outcomes

The analysis of the *WHO-5 Well-Being Index* (primary outcome) indicated significant group differences in quality of life at week 4 in favour of the modified fasting group (mean difference (MD) 5.93; 95% confidence interval (CI), 0.02–11.84; *p* < 0.05) (Table 2); no effects were found at 6 months follow-up.

**Table 2:** Means and estimated group differences in subjective questionnaire data between Modified fasting and Fasting as usual

	**Modified fasting** **mean ± standard deviation**			**Fasting as usual** **mean ± standard deviation**			**Estimated** **group difference** **(95% confidence interval)**	***p*-value**	**Estimated** **group difference** **(95% confidence interval)**	***p*-value**
	**Baseline**	**Week 4**	**6 months**	**baseline**	**Week 4**	**6 months**	**Week 4**		**6 months**	
Quality of life (WHO-5)^b^	59.4 ± 15.7	67.1 ± 12.7	63.8 ± 15.3	54.2 ± 16.8	60.5 ± 19.6	61.2 ± 18.5	5.9 (0.02; 11.8)^a^	0.049	1.09 (-4.8; 7.0)	0.715
BMLSS-Life	72.7 ± 14.2	75.6 ± 14.2	74.4 ± 17.0	71.1 ± 12.9	76.9 ± 12.6	72.2 ± 14.7	-1.3 (-5.27; 2.74)	0.534	0.76 (-3.9; 5.4)	0.746
BMLSS-Health	67.7 ± 18.8	72.6 ± 17.3	67.3 ± 17.8	68.5 ± 16.5	67.7 ± 18.6	65.4 ± 18.6	5.9 (0.19; 11.67)	0.043	2.99 (-2.8; 8.8)	0.309
CPSC-Mindfulness	57.9 ± 14.8	63.2 ± 14.5	60.9 ± 16.5	60.3 ± 15.3	58.0 ± 16.3	56.5 ± 14.6	7.6 (2.68; 12.52)	0.003	5.40 (0.2; 10.6)	0.043
ERG-Openness	72.4 ± 20.0	76.5 ± 18.0	75.2 ± 17.9	73.2 ± 17.9	73.6 ± 16.0	73.0 ± 15.3	3.8 (-0.69; 8.28)	0.096	2.13 (-2.7; 7.0)	0.382
ERG-Easiness	56.2 ± 9.7	58.9 ± 10.3	55.7 ± 12.6	53.8 ± 12.7	54.8 ± 11.0	54.1 ± 10.2	4.1 (0.38; 7.80)	0.031	0.30 (-3.8; 4.4)	0.884
SPREUK-CRP	67.8 ± 16.4	70.3 ± 19.3	64.3 ± 19.0	69.1 ± 19.6	72.6 ± 18.9	63.9 ± 18.8	-1.1 (-6.34; 4.20)	0.687	1.54 (-3.4; 6.5)	0.540
SPREUK-HP	74.0 ± 12.9	77.9 ± 14.1	72.6 ± 14.0	78.8 ± 13.1	77.9 ± 14.0	73.8 ± 16.3	3.0 (-1.11; 7.14)	0.150	1.09 (-3.8; 5.9)	0.657
SPREUK-URP	32.7 ± 13.8	39.9 ± 19.1	38.4 ± 18.6	37.6 ± 16.2	43.9 ± 18.8	39.9 ± 18.9	-0.1 (-6.27; 6.15)	0.985	2.10 (-3.7; 7.9)	0.476
SPREUK-NAT	61.2 ± 18.6	61.6 ± 19.5	63.9 ± 16.9	57.3 ± 21.7	58.8 ± 21.2	57.3 ± 22.9	-0.6 (-6.24; 5.08)	0.839	4.71 (-1.6; 11.0)	0.139
SPREUK-EXIST	65.1 ± 18.4	70.0 ± 19.2	64.6 ± 18.1	68.8 ± 19.2	74.3 ± 21.0	67.7 ± 22.7	-1.5 (-6.86; 3.95)	0.595	-0.30 (-6.2; 5.6)	0.920
SPREUK-GRAT	73.1 ± 19.2	76.5 ± 18.9	72.4 ± 18.9	75.1 ± 17.4	76.2 ± 17.2	73.1 ± 21.1	2.0 (-3.29; 7.24)	0.458	-0.65 (-7.1; 5.8)	0.840
PROMIS-SD	20.9 ± 6.8	18.4 ± 6.1	19.0 ± 6.3	21.1 ± 6.6	20.0 ± 7.0	20.1 ± 6.3	-2.2 (-4.53; 0.04)	0.055	-1.22 (-3.2; 0.8)	0.224
PROMIS-SRI	18.8 ± 7.0	17.1 ± 5.9	17.0 ± 6.0	20.5 ± 6.8	20.9 ± 8.2	18.2 ± 6.6	-3.5 (-6.04; -1.03)	0.006	-0.32 (-2.6; 1.9)	0.778
FACIT-Peace	70.8 ± 15.2	77.0 ± 12.4	74.2 ± 11.7	68.6 ± 14.0	73.2 ± 11.3	69.2 ± 15.2	3.3 (-0.58; 7.19)	0.094	3.76 (-0.7; 8.2)	0.094
FACIT-Meaning	88.8 ± 7.9	91.8 ± 7.0	91.0 ± 7.6	88.7 ± 10.9	90.8 ± 8.6	86.0 ± 12.0	0.8 (-1.66; 3.24)	0.522	4.74 (1.2; 8.2)	0.008

Group differences are estimates from the ANCOVA with 95% confidence intervals (CI)

*BMLSS* Brief Multidimensional Life Satisfaction Scale, *CPSC* Conscious Presence and Self Control, *FACIT* Functional Assessment of Chronic Illness Therapy, *PROMIS-SD* Sleep Disturbance, *PROMIS-SRI* Sleep Related Impairment, *SPREUK* Spiritual and Religious Attitudes in Dealing with Illness (subscales: *CRP* Frequency of Engagement in Spiritual Practices, *HP* Frequency of Engagement in Humanistic Practices, *URP* Frequency of Engagement in Unspecific Spiritual Practices, *NAT* Frequency of Engagement in Nature Oriented Practices, *EXIST* Frequency of Engagement in Existentialistic Practices, *GRAT* Frequency of Engagement in Gratitude and Awe), *WHO* World Health Organization

^a^*p* < 0.05

^b^primary outcome

At week 4, group differences were detected in situational awareness/mindfulness (CPSC) (MD 7.6; 95% CI 2.68–12.52), positive life construction (ePLC) (MD 4.1; 95% CI, 0.38–7.80), and overall satisfaction with health (BMLSS-10) (MD 5.9; 95% CI, 0.19–1.67) in favour of the modified fasting group. Long-term follow-up showed group differences for mindfulness (MD 5.40; 95% CI, 0.20–10.6), and the meaning subscale of the FACIT-Sp (MD 4.74; 95% CI, 1.20–8.2) (Table 2). There were no differences between groups for other well-being outcomes, including sleep and engagement in spiritual activities (Table 2).

### Anthropometric Measures and Blood Biomarkers

Group differences in favour of the modified fasting group were found for weight (MD − 0.9; 95% CI, − 1.39 to − 0.42), BMI (MD − 0.3; 95% CI, − 0.15 to − 0.50), hip circumference (MD − 1.3; 95% CI, − 2.32 to − 0.37), and diastolic blood pressure (MD − 2.8; 95% CI, − 5.15 to − 0.43) at week 4 (Table 3). While the majority of parameters remained relatively stable, there was an observed increase in average blood pressure and heart rate over the duration of the study, with the highest values recorded after 6 months.

**Table 3:** Means and estimated group differences in anthropometric data between Modified fasting and Fasting as usual

	**Modified fasting** **mean ± standard deviation**			**Fasting as usual** **mean ± standard deviation**			**Estimated** **group difference** **(95% confidence intervals)**	***p*-value**	**Estimated** **group difference** **(95% confidence intervals)**	***p*-value**
	Baseline	Week 4	6 months	Baseline	Week 4	6 months	Week 4		6 months	
Weight, kg	75.2 ± 14.5	72.1 ± 14.0	74.4 ± 15.9	74.9 ± 14.7	72.8 ± 14.4	75.0 ± 14.5	-0.9 (-1.39; -0.42)	< 0.001	-0.96 (-2.4; 0.5)	0.188
BMI, kg/m^2^	26.2 ± 4.3	25.1 ± 4.2	25.9 ± 4.8	25.6 ± 4.3	24.9 ± 4.2	25.7 ± 4.2	-0.3 (-0.50; -0.15)	< 0.001	-0.34 (-0.8; 0.2)	0.178
Muscle mass, %	31.5 ± 10.3	31.0 ± 6.6	30.5 ± 6.6	30.8 ± 6.5	30.0 ± 6.3	30.4 ± 6.4	1.0 (-0.15; 2.15)	0.087	0.17 (-0.8; 1.1)	0.732
Fat mass, %	32.3 ± 9.8	30.9 ± 9.3	32.6 ± 9.1	31.7 ± 9.5	31.6 ± 9.0	32.4 ± 9.3	-1.0 (-2.03; 0.07)	0.066	-0.16 (-1.1; 0.8)	0.743
Visceral fat mass, %	7.1 ± 3.9	6.4 ± 4.1	7.1 ± 4.1	6.3 ± 3.0	5.8 ± 3.0	6.6 ± 3.2	-0.2 (-0.61; 0.27)	0.445	-0.23 (-0.6; 0.2)	0.246
Waist circumference, cm	89.9 ± 10.5	86.9 ± 11.9	90.9 ± 11.6	88.5 ± 9.7	86.7 ± 11.2	90.4 ± 10.4	-1.0 (-2.48; 0.49)	0.185	-0.85 (-2.5; 0.8)	0.313
Hip circumference, cm	105.8 ± 7.8	103.4 ± 7.9	105.9 ± 7.8	105.7 ± 9.0	104.7 ± 8.9	106.9 ± 8.5	-1.3 (-2.32; -0.37)	0.007	-1.06 (-2.1; 0.01)	0.052
Waist to hip ratio	0.8 ± 0.1	0.8 ± 0.1	0.9 ± 0.1	0.8 ± 0.1	0.8 ± 0.1	0.8 ± 0.1	0.0 (-0.01; 0.01)	0.730	0.00 (-0.0; 0.0)	0.920
Systolic blood pressure, mmHg	113.7 ± 14.6	114.7 ± 12.6	117.6 ± 13.8	113.6 ± 11.7	114.4 ± 11.7	115.1 ± 13.0	-0.2 (-3.71; 3.38)	0.927	1.79 (-1.7; 5.3)	0.317
Diastolic blood pressure, mmHg	71.3 ± 9.6	71.5 ± 8.5	74.6 ± 9.7	71.7 ± 8.3	73.8 ± 8.5	72.1 ± 9.3	-2.8 (-5.15; -0.43)	0.021	1.89 (-1.0; 4.7)	0.191
Heart rate, bpm	72.1 ± 8.3	69.5 ± 9.6	75.8 ± 10.5	73.0 ± 11.1	69.6 ± 10.4	75.9 ± 8.8	0.3 (-2.62; 3.19)	0.846	0.66 (-2.7; 4.0)	0.699

Group differences are estimates from the ANCOVA with 95% confidence intervals (CI)

There were no differences identified between groups for the blood serum markers after the intervention (Table 4). There were also no differences between groups in anthropometric parameters or serum markers for the long-term follow-up (Tables 3 and 4). Although most parameters remained stable, the data revealed a significant increase in cholesterol, LDL-cholesterol, and HDL-cholesterol levels following the fasting period. Conversely, markers of liver function demonstrated a decrease during the same period.

**Table 4:** Means and estimated group differences in blood serum biomarkers between the Modified fasting and Fasting as usual

	**Modified fasting** **mean ± standard deviation**		**Fasting as usual** **mean ± standard deviation**		**Estimated group difference** **(95% confidence intervals)**	***p*-value**
	Baseline	Week 4	Baseline	Week 4	Week 4	
Triglycerides, mg/dL	77.1 ± 40.3	69.9 ± 38.2	76.1 ± 44.0	67.4 ± 29.6	-0.2 (-9.86; 9.44)	0.956
Cholesterol, mg/dL	181.8 ± 34.0	189.8 ± 36.8	175.0 ± 31.9	186.8 ± 31.3	4.9 (-2.41; 12.15)	0.187
HDL-cholesterol, mg/dL	57.1 ± 14.0	60.3 ± 15.2	59.2 ± 14.5	64.2 ± 15.9	-2.1 (-4.90; 0.78)	0.154
LDL-cholesterol, mg/dL	110.8 ± 29.9	122.4 ± 32.7	103.0 ± 28.9	117.3 ± 29.1	-1.7 (-7.67; 4.23)	0.567
AST, U/L	24.7 ± 6.1	20.7 ± 5.4	24.3 ± 10.0	20.0 ± 5.1	0.5 (-1.15; 2.21)	0.532
ALT, U/L	25.8 ± 11.4	22.9 ± 12.6	23.4 ± 13.2	20.8 ± 10.4	0.3 (-2.15; 2.81)	0.792
GGT, U/L	20.2 ± 18.3	17.0 ± 16.3	17.1 ± 10.9	14.4 ± 7.6	-0.2 (-1.33; 1.00)	0.781
FBG, mg/dL	85.6 ± 10.5	76.9 ± 5.2	84.3 ± 6.0	77.4 ± 5.2	-1.0 (-2.84; 0.76)	0.253
Uric acid, mg/dL	4.7 ± 1.5	4.9 ± 1.3	4.7 ± 1.4	4.8 ± 1.2	0.1 (-0.12; 0.33)	0.342
Creatinine, mg/dL	0.6 ± 0.2	0.8 ± 0.2	0.6 ± 0.2	0.8 ± 0.2	0.0 (-0.04; 0.04)	0.937

Group differences are estimates from the ANCOVA with 95% confidence intervals (CI)

*ALT* alanine aminotransferase, *AST* aspartate aminotransferase, *FBG* fasting blood glucose, *GGT* gamma-glutamyltransferase

### Safety

Overall, 35 participants (57.9%) in the Modified fasting group and 31 participants (60.0%) in the Fasting as usual group reported adverse events. Adverse events in the modified fasting group included headaches and/or migraine (*n* = 15), dizziness and/or fatigue (*n* = 7), common cold (*n* = 9), gastrointestinal problems (diarrhoea, pain, nausea) (*n* = 6), and one case each of hay fever, back pain, joint pain, atopic eczema, fungal infection, tonsillitis, cystitis, and superficial skin burn. Adverse events in the Fasting as usual group included headaches and/or migraines (*n* = 14), dizziness and/or fatigue (*n* = 9), common cold (*n* = 4), gastrointestinal problems (diarrhoea, pain, nausea) (*n* = 5), and one case each of fainting, atopic eczema, and dry skin. All adverse events were temporary, and most were unrelated to the dietary and lifestyle modifications, not however to fasting generally.

Several participants showed elevated uric acid levels, including at baseline (7 in modified fasting, 4 in fasting as usual) as well as after Ramadan (6 in modified fasting, 2 in fasting as usual) (Table 4). None of the participants reported any physical complaints due to the elevated uric acid levels, and they were rated as not clinically significant. Nevertheless, all participants were advised to consult with their GP for further observation.

Two serious adverse events were recorded but they were found to be unrelated to the intervention — one participant in the modified fasting group was hospitalized after experiencing an allergic shock but fully recovered soon after, and one participant in the control group experienced a vertebral disc prolapse and received separate medical treatment.

### Adherence

Nearly all participants in the modified fasting group (*n* = 55, 96.5%) reported being able to follow the recommendations, with an average of 74.9% (SD = 14.5) of the recommendations followed. Recommendations that were easily followed were to have smaller meals (*n* = 46, 80.7% of respondents), consume less sugar (incl. soft drinks) (*n* = 44, 77.2%), eat more vegetables (*n* = 49, 86%), and drink more water (*n* = 44, 77.2%). Fourteen participants interrupted their consecutive fasting days due to health concerns, including adverse events experienced by 2 participants in each group. Women also interrupted the fasting during their menstruation for an average of 4.9 days. Average days of broken fast were 7.0 days for women and 1.2 days for men.

### Overall Satisfaction

All but 15 participants (9 men, 6 women) in the modified fasting group reported subjective, self-observed changes compared to their usual Ramadan fasting experience, from increased overall mental well-being (*n* = 10, 17.5%), to being more lively and fit (*n* = 15, 26.3%), less feelings of fullness and/or bloating (*n* = 9, 15.8%), and decreased feelings of appetite (*n* = 4, 7.0%) and thirst (*n* = 2, 3.5%) during the day. Overall, 54 out of 57 (94.7%) reported that they would recommend the modified fasting approach to their family and friends.

## Discussion

### Principal Findings

In this study, a pre-Ramadan dietary and lifestyle modification increased quality of life of healthy adult Muslims during Ramadan fasting. Benefits over fasting as usual were also found for satisfaction with health, ease of life and mindfulness, as well as body weight, BMI, hip circumference, and diastolic blood pressure. Effects were observed directly after Ramadan, but not after 6 months. Two out of three participants reported transient adverse events, most commonly headaches/migraines, dizziness/fatigue, common cold, and gastrointestinal symptoms, with no differences between groups. Satisfaction with the dietary and lifestyle modifications was very high.

### Well-Being Outcomes

This is the first randomised controlled trial to examine the effects of a modified Ramadan fasting regimen on quality of life and well-being. The overall effects of Ramadan on well-being might not only be related to diet and lifestyle, but to spirituality and sociocultural connection, which are recognized predictors of quality of life and well-being [79] and may serve as important psychosocial resources for coping with stress, depression, and anxiety [80, 81]. Traditionally, Ramadan fasting is concerned with the cultivation of positive attributes such as purification and ‘God-consciousness’ (Quran 2:183). In a study of healthy Muslim graduate students, Bayani et al. [82] found that Ramadan fasting promoted overall psychological well-being, self-acceptance, autonomy, positive relations, environmental mastery, and personal growth. During the COVID-19 pandemic, Ramadan fasting was found to improve mental well-being among healthy adult Muslims in Nigeria [63] and Iran [83]. To further demonstrate subjective benefits, in some people with chronic illnesses, the benefits to quality of life and well-being afforded by participating in Ramadan can outweigh the physical challenges or disease exacerbations associated with abstinence from food, water, and medications [37, 40, 84, 85].

Nonetheless, research and anecdotal evidence suggests that some observers experience little to no benefit to well-being (or subjective aspects thereof) under fasting as usual. For example, Nugraha et al. [86] found hardly any benefit to quality of life and mental health in the fasting group compared to the non-fasting group, while sleepiness and depression were significantly higher in the fasting group between week 3 and week 4 of Ramadan. These groups however might not be comparable, and differ significantly with regard to their psychological profiles, including faith, self-efficacy, and coping. In another study of healthy adult men, Harder-Lauridsen et al. [87] found a significant reduction in positive feelings in the afternoon during Ramadan compared to the normal control period. Ramadan fasting has been linked to increased irritability [22, 88], reduced mental alertness [27], impaired memory [89], decreased positive mood [87, 90], and feelings of fatigue [22, 90, 91] in healthy people, as well as depressive symptoms and decreased quality of life in people with metabolic, gastrointestinal, and respiratory diseases [61, 92–95]. A review by Cherif et al. [96] concludes that mental health, including coping strategies and decision-making abilities, may be adversely affected by Ramadan fasting. Considering the positive outcomes on well-being for the participants in our study, this shows there is a need for dietary and lifestyle interventions appropriate to the local culture to inform Ramadan observers to ameliorate potential declines in mental health and well-being.

### Physical Health

In this study, small reductions in body weight, BMI, hip circumference, and diastolic blood pressure were found in favour of the modified Ramadan fasting regimen. The weight loss was more than was found in a meta-analysis conducted by Correia et al. [29], who reported an average weight loss of − 0.35 kg in Ramadan observers, there was however substantial variation between the included studies owing to geographical, cultural, and sociodemographic characteristics.

Increased access to high-energy, ultra-processed food, and decreased physical activity have contributed to an increasing prevalence of ill-health in the general population [97], particularly in the Muslim population [98, 99]. These behaviours were exacerbated during Ramadan, in some groups more so than others [2, 14, 19, 100]. Shadman et al. [19] for example identified major Ramadan dietary patterns throughout Tehran, the least healthy of which were the [1] Western-like pattern — high in fast foods, salty snacks, nuts, potato, meat, chocolates, and juices and; [2] high-cholesterol and high-sweet junk food pattern — high in pickles, sweets and condiments, butter and cream, canned fish, visceral meats, and eggs; these diets were also associated with less physical activity and a high prevalence of obesity. During Ramadan, significantly higher than normal sugar and carbohydrate intake has been reported in Lebanese [14] and Saudi Arabian [16, 17] communities, while relatively healthy Ramadan diets have been reported in studies from Africa [18, 20] and Iran [101]. Such variability in Ramadan diets explains, at least in part, the variability in physical health parameters reported in the literature [5–13] and may also contribute to variability in personal satisfaction and feelings of overall well-being.

Notwithstanding, despite the immediate effects of Ramadan fasting on body weight, these effects appear transient and reversible: after the end of Ramadan, we observed a return to the pre-Ramadan condition, as noted in this study and others [7, 12, 29]. Potentially, the prescription of healthy diet and lifestyle maintenance strategies post-Ramadan could be valuable for the preservation of long-term health and warrants further exploration.

### Satisfaction and Safety

Overall, participants in this study reported a high degree of satisfaction with the modified fasting regimen. Minor, transient adverse events (headache, fatigue, gastro-intestinal complaints) are typical of intermittent fasting [102] and were observed in both groups equally. Despite similar adverse event patterns, participants in the modified fasting group reported improved mental well-being compared to other fasting periods, and better physical health during Ramadan for example by feeling less full or bloated and feeling less appetite and thirst during the day. While a modified Ramadan fasting can be considered safe, this may not necessarily apply to older and less healthy populations.

The increases in cholesterol found in both groups over time can be related to the hydration status of the sample, who would have been fasting before the post-intervention blood draw, conducted right before breaking the fast for the day. The increase in blood pressure and heart rate, specifically after 6 months, might warrant further investigation, but it would have fallen into December of 2016, and it is likely that the winter season may have impacted blood pressure and heart rate.

### Strengths and Limitations

This is the first study applying a dietary and lifestyle modification to a religious practice, investigating the effects of modified Ramadan fasting on quality of life, and well-being. As such the design may serve as a successful example for investigating the health benefits of religious practices where the usual RCT design might be unethical, given some religious practices are mandatory. The study further had very little attrition and found high levels of adherence to study procedures and the intervention.

Despite its strengths, this study has several limitations. The study sample skewed towards a younger, more educated, and healthier demographic, likely influenced by the recruitment strategy. Additionally, language barriers may have hindered participation among mid-aged and older Muslims who did not grow up in Germany. As a result, the effects of dietary and lifestyle modifications might have been smaller than they would have in older, less healthy adults, as such they cannot be generalised to the general population. We also did not investigate individual diets by means of food diaries or questionnaires, as such the changes in diet during the trial could not be quantified. In addition, daily activity was not monitored, and as such no inference about the effects of daily activity on well-being, and safety could be drawn.

At the same time, this particular Ramadan was carried out around the summer solstice with days nearly 17 h long, and day temperatures ranging from 18 to 32 °C in Essen, Germany, making it one of the most challenging fasting periods for Ramadan observers in this part of the world. The trial was conducted in 2016. Despite the passage of time, it is important to note that the observance of Ramadan has remained consistent over the past decade. Thus, interventions such as those investigated in this study continue to hold contemporary significance and relevance.

Notwithstanding, differences in the ways that Ramadan is practiced in different regions and seasons among different cultures make it difficult to generalise to all of those who partake, which is widely acknowledged [2, 5, 11].

### Implications for Future Research

Further studies could be conducted in other geographical and cultural settings to validate the findings and adapt the intervention to the local needs of Ramadan observers. Research undertaken in regions known for generally poorer diet and lifestyle behaviours (e.g. Southeast Asia, Saudi Arabia, and USA [103–105]) and targeted recruitment of at-risk groups (e.g. low socioeconomic status, those with overweight/obesity [106]) is important since the outcomes on health and well-being likely have a greater impact. Future studies could also be improved by including detailed food and nutrition, activity, and sleep diaries to relate these data directly to health and well-being. Future studies might also consider more tailored health and lifestyle advice. Finally, understanding the cumulative benefits of healthier successive annual Ramadan periods, and the long-term uptake of post-Ramadan health maintenance education is warranted.

## Conclusion

Pre-Ramadan dietary and lifestyle advice can lead to short-term improvements in mental and physical well-being of healthy adult Muslims during Ramadan. As such, this study demonstrates the potential benefits of culturally appropriate dietary and lifestyle interventions in religious contexts. Furthermore, our methodology may provide a successful example for investigating the health benefits religious practices where usual RCT designs might be unethical, or impractical.

## Data Availability

The participants of this study did not give written consent for their data to be shared publicly, so due to the sensitive nature of the research supporting data is not available.
